# Electrocardiographic manifestations in a large right-sided pneumothorax

**DOI:** 10.1186/s12890-021-01470-1

**Published:** 2021-03-23

**Authors:** Hiroyuki Yamamoto, Kazuhiro Satomi, Yoshiyasu Aizawa

**Affiliations:** 1 Department of Cardiovascular Medicine, Narita-Tomisato Tokushukai Hospital, 1-1-1 Hiyoshidai, Tomisato, Chiba 286-0201 Japan; 2 Department of Cardiology, Tokyo Medical University, Tokyo, Japan; 3 Department of Cardiovascular Medicine, International University of Health and Welfare, Chiba, Japan

**Keywords:** Right-sided pneumothorax, Chest pain, Electrocardiogram, Phasic voltage variation, P-pulmonale, Vertical P-wave axis

## Abstract

**Background:**

Pneumothorax is an extrapulmonary air accumulation within the pleural space between the lung and chest wall. Once pneumothorax acquires tension physiology, it turns into a potentially lethal condition requiring prompt surgical intervention. Common symptoms are chest pain and dyspnea; hence an electrocardiogram (ECG) is often performed in emergent settings. However, early diagnosis of pneumothorax remains challenging since chest pain and dyspnea are common symptomatology in various life-threatening emergencies, often leading to overlooked or delayed diagnosis. While the majority of left-sided pneumothorax-related ECG abnormalities have been reported, right-sided pneumothorax-related ECG abnormalities remain elucidated.

**Case presentation:**

A 51-year-old man presented to the emergency department with acute-onset chest pain and dyspnea. Upon initial examination, the patient had a blood pressure of 98/68 mmHg, tachycardia of 100 beats/min, tachypnea of 28 breaths/min, and oxygen saturation of 94% on ambient air. Chest auscultation revealed decreased breath sounds on the right side. ECG revealed sinus tachycardia, phasic voltage variation of QRS complexes in V4–6, P-pulmonale, and vertical P-wave axis. Chest radiographs and computed tomography (CT) scans confirmed a large right-sided pneumothorax. The patient’s symptoms, all the ECG abnormalities, and increased heart rate on the initial presentation resolved following an emergent tube thoracostomy. Moreover, we found that these ECG abnormalities consisted of two independent factors: respiratory components and the diaphragm level. Besides, CT scans demonstrated the large bullae with a maximum diameter of 46 × 49 mm in the right lung apex. Finally, the patient showed complete recovery with a thoracoscopic bullectomy.

**Conclusions:**

Herein, we describe a case of a large right-sided primary spontaneous pneumothorax with characteristic ECG findings that resolved following re-expansion of the lung. Our case may shed new light on the mechanisms underlying ECG abnormalities associated with a large right-sided pneumothorax. Moreover, ECG manifestations may provide useful information to suspect a large pneumothorax or tension pneumothorax in emergent settings where ECGs are performed on patients with acute chest pain and dyspnea.

**Supplementary Information:**

The online version contains supplementary material available at 10.1186/s12890-021-01470-1.

## Background

Pneumothorax is defined as an abnormal air trapping within the pleural space between the lung and the chest wall. Primary spontaneous pneumothorax (PSP) occurs in approximately 7 per 100,000 men and 1 per 100,000 women per year [1]. It is most commonly associated with smoking, tall and thin body habitus, Marfan syndrome, pregnancy, or familial history of pneumothorax. PSP is most frequently diagnosed between 20 and 30 years of age. Common clinical symptoms are chest pain and dyspnea. The conversion from spontaneous pneumothorax to tension pneumothorax (TPT) results in a shift of the mediastinal structures to the unaffected side with tension physiology, leading to acute respiratory failure and severe hemodynamic compromise with potentially fatal sequelae [2]. Diagnosis can be confirmed by multimodality imaging such as chest radiograph, computed tomography (CT), or pleural ultrasonography. However, given the potential for various life-threatening emergencies such as acute coronary syndrome, acute aortic dissection, acute pulmonary embolism, or acute pericarditis, the correct early diagnosis of pneumothorax with common symptomatology of chest pain and dyspnea remains extremely challenging [3]. Thus, a simple and useful screening tool for diagnosing pneumothorax is required to avoid unfavorable outcomes.

The 12-lead electrocardiogram (ECG) is readily available during initial patient evaluation with chest pain and dyspnea in emergent settings. Although many left-sided pneumothorax-associated ECG abnormalities and their possible mechanisms have been mentioned [4], most right-sided pneumothorax-associated ECG abnormalities remain ambiguous.

## Case presentation

A 51-year-old man presented to the emergency department with acute-onset chest pain and dyspnea. Upon initial examination, the patient had a blood pressure of 98/68 mmHg, tachycardia of 100 beats/min, tachypnea of 28 breaths/min, and oxygen saturation of 94% on ambient air. He was agitated with cold sweats. Cardiac auscultation revealed tachycardia without cardiac murmurs. Chest auscultation revealed decreased breath sounds on the right side. Neither jugular vein distension nor lower extremities edema was observed. He had a history of hypertension and had smoked 15 cigarettes daily for 35 years.

**Fig. 1:**
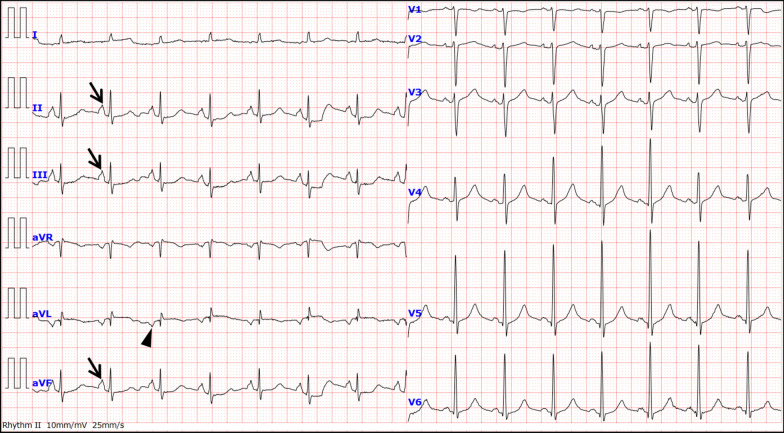
Electrocardiogram. Electrocardiogram reveals sinus tachycardia of 100 beats/min and prominent phasic voltage variation of QRS complexes in precordial leads (V4–6). The P-waves’ abnormally high amplitude (arrows, maximum 3.0 mm in voltage, reference: < 2.5 mm) in the inferior leads, and vertical P-wave axis are observed. The P-wave inversion in aVL (arrowhead) is visible

**Fig. 2:**
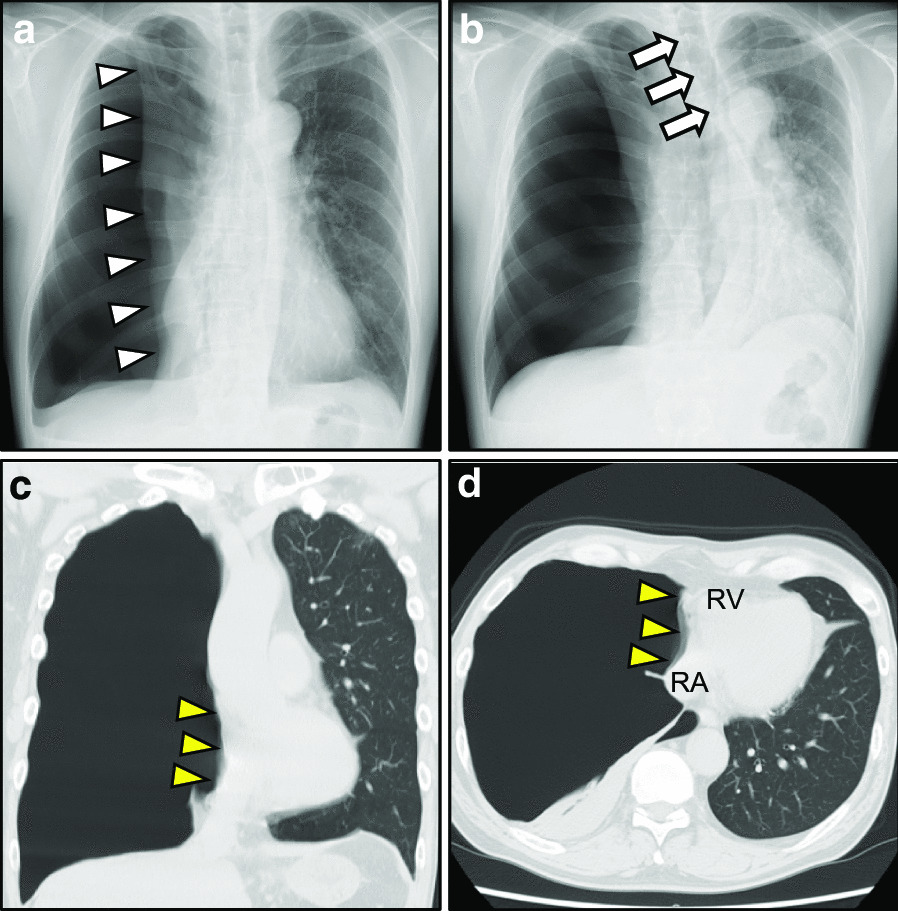
Initial chest radiographs and computed tomography scans (**a**, **c**, and **d**: during inspiration, **b**: during expiration). **a** Chest radiographs reveal a huge right-sided pneumothorax during inspiration. The absence of lung markings peripheral to the thin white visceral pleural line (white arrowheads) can be noted. **b** During expiration, increased intercostal space and a pronounced leftward mediastinal shift (arrows) are recognized. The hemidiaphragm on the right side remains quite depressed. **c**, **d** Chest computed tomography scans reveal a huge right-sided pneumothorax during inspiration. The extracardiac compression of the right atrial free wall result in a straightened cardiac border (yellow arrowheads). *RA* right atrium, *RV* right ventricle

ECG revealed sinus tachycardia of 91 beats/min, a vertical P-wave axis of 97 degrees, normal QRS axis of 55 degrees, P-pulmonale, and phasic voltage variation (PVV) of QRS complexes in V4–6 (Fig. 1). Each respiratory component (inspiration/expiration) characterized further ECG abnormalities (Table 1; Additional file 1). Note the prominent increase of QRS amplitudes in V4–6 during expiration. However, all the P-pulmonale, vertical P-wave axis, and tachycardia remained unchanged regardless of the inspiration–expiration cycle. Echocardiography revealed normal LV contraction with trivial tricuspid regurgitation, indicating less likely pulmonary hypertension. Chest radiographs and CT scans revealed a large right-sided pneumothorax (Fig. 2). Note the straitened cardiac border in the right atrium (RA) and the trachea’s prominent leftward deviation during expiration. Laboratory testing revealed that both cardiac enzymes and brain natriuretic peptide levels were within the normal ranges.

**Table 1:** Respiratory comparison in the ECG variables of the pre- and post- treatment of tube thoracostomy

ECG variables	Inspired	Expired
Pre HR (bpm)	91	88
Post HR (bpm)	54	56
Pre PR interval (ms)	148	138
Post PR interval (ms)	148	156
Pre QRS duration (ms)	86	88
Post QRS duration (ms)	88	86
Pre QTc interval (ms)	429	426
Post QTc interval ( ms )	417	419
Pre P-wave axis (°)	92	98
Post P-wave axis (°)	23	43
Pre QRS axis (°)	62	40
Post QRS axis (°)	64	22
Pre T-wave axis (°)	70	69
Post T-wave axis (°)	63	42
Pre RV5 (mV)	1.5	3.14
Post RV5 (mV)	1.29	1.65
Pre SV1 (mV)	1.08	0.76
Post SV1 (mV)	1.19	1.26
Pre RV5 + SV1 (mV)	2.59	3.9
Post RV5 + SV1 (mV)	2.48	2.91
Pre PII (mV)	3	3
Post PII (mV)	1	1

*ECG* electrocardiogram, *HR* heart rate, *QTc* corrected QT, *RV5* R-wave voltage in V5, *SV1* S-wave voltage in V1, *PII* P-wave voltage in II, *bpm* beats/min

**Fig. 3:**
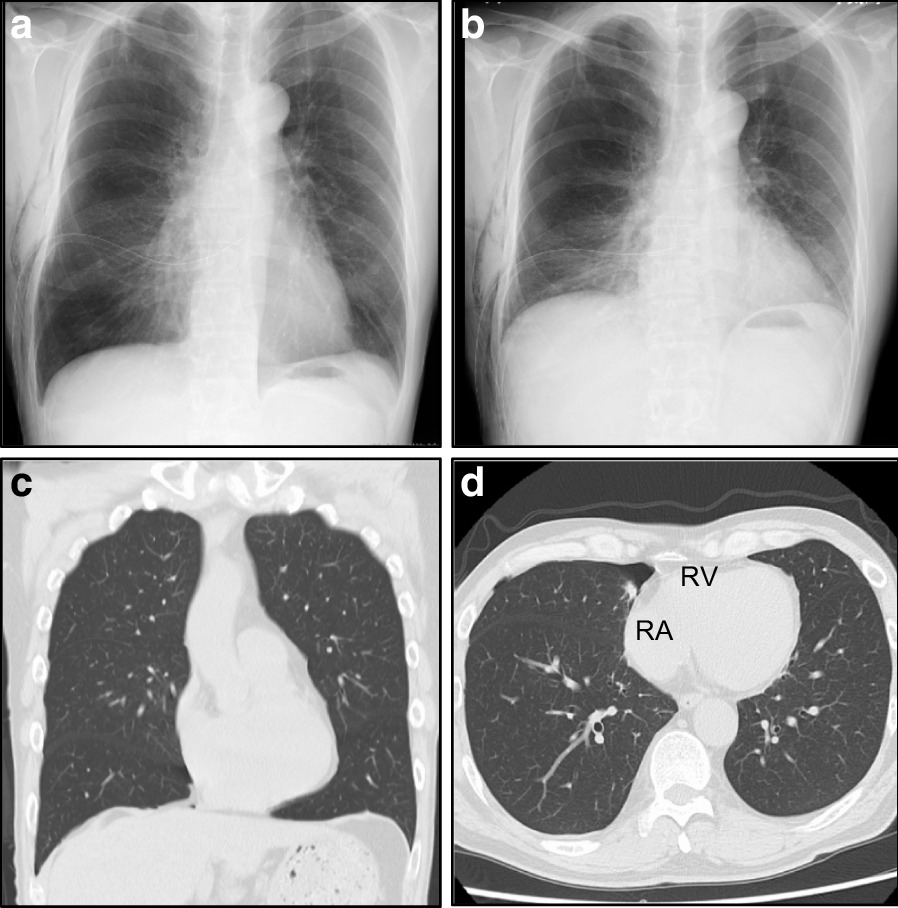
Follow-up chest radiographs and computed tomography scans of the post-tube thoracostomy (**a**,** c**,** d**: during inspiration, **b**: during expiration). **a**–**d** Follow-up chest radiographs and computed tomography scans reveal the resolution of pneumothorax. The resolution of right atrial collapse can be noted. *RA* right atrium, *RV* right ventricle

**Fig. 4:**
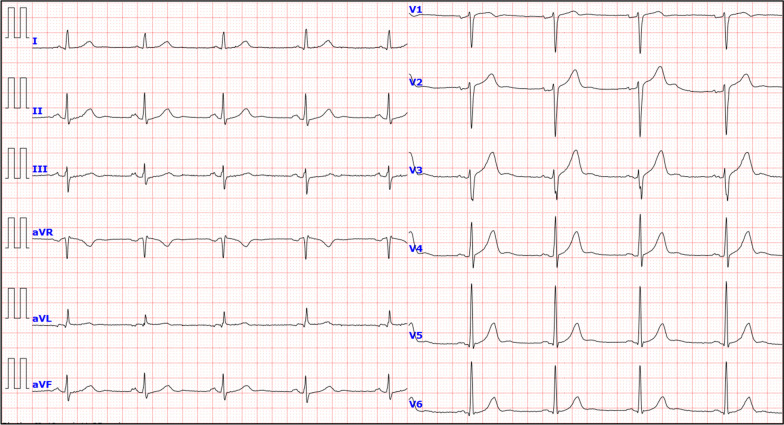
A follow-up electrocardiogram (ECG) following the tube thoracostomy. A follow-up ECG after the tube thoracostomy revealed normalization of increased heart rate and the resolution of all the ECG abnormalities recognized on initial ECG

An emergent tube thoracostomy was performed for the pneumothorax. Immediately after the procedure, the patient’s symptoms resolved. The follow-up chest radiographs and CT scans revealed the lungs’ re-expansion and resolution of the RA deformity (Fig. 3). Strikingly, the follow-up ECG demonstrated that all the ECG abnormalities and increased heart rate on the initial presentation were resolved (Fig. 4). Moreover, we confirmed that no ECG abnormalities were induced during inspiration or expiration following the lungs’ re-expansion (Table 1; Additional file 2). In addition, CT scans demonstrated multiple bullae in the lung apices. Note the large bullae with a maximum diameter of 46 × 49 mm in the right lung apex (Additional file 3), suggesting a PSP’s potential cause. Finally, the patient underwent a thoracoscopic bullectomy on day 21. The resected specimen’s pathological examination confirmed multiple bullae with pleural fibrosis and thickening and no evidence of malignancy. The postoperative course was uneventful. On day 23, the patient was discharged with normal vital signs: blood pressure, 134/87 mmHg; heart rate, 70 beats/min; respiratory rate, 14 breaths/min; and oxygen saturation, 98% on ambient air. The patient remained symptom-free during the follow-up.

## Discussion and conclusions

The majority of ECG abnormalities reported in patients with pneumothorax are related to left-sided pneumothorax; right-axis deviation, clockwise rotation of the transition zones, inverted T-waves, and diminution of QRS-wave amplitude in the precordial leads [5]. These ECG changes have been explained primarily by the changes in the heart’s anatomic position within the thoracic cavity. Possible mechanisms include several factors affecting electrical impulses; the rightward shift of the mediastinum, the clockwise rotation of the heart, and the intrusion of electrically insulated extrapulmonary air accumulation between the heart and the electrodes. Besides, the increased pulmonary vascular resistance caused by the increased intrathoracic pressure may be involved too. On the other hand, although less frequent, various ECG changes related to right-sided pneumothorax have been reported, whose mechanisms remain poorly understood [5–7].

We report a case of distinct ECG manifestations of a large right-sided PSP. Our case may provide several valuable clinical lessons.

First, right-sided PSP can present as PVV on ECG.

Previous reports have suggested that PVV on ECG is a specific sign of left-sided large pneumothorax, or TPT [8–12]. It has been speculated that the heart’s periodic movement within the thorax caused by beat-to-beat or respiration was involved in this ECG change [10, 13]. Notably, the R-wave voltages in V4–6 on ECG are shortened during inspiration and augmented during expiration in our case. While the upper movement of the diaphragm normally results in an upward shift of the heart during expiration, in our case, the increased intrathoracic pressure deviates the mediastinal structures to the left side of the chest cavity during expiration, causing the heart to shift further up-left. Consequently, the left lateral electrodes are even closer to the heart and receive the maximum cardiac electrical potential (greater R-wave voltage in V4–6), resulting in what appears to be left ventricular hypertrophy. These findings support the hypothesis that respiratory components are the main contributors to the mechanism underlying PVV on ECG in the right-sided pneumothorax, similar to that in left-sided pneumothorax. Moreover, while longer ECG recordings during normal respiration revealed PVV of the QRS complexes in V4–6 on admission, none of the longer ECG recordings during either inspiration or expiration revealed any cyclic variation of the QRS complexes in V4–6 (Additional file 4), thus confirming the concept of respiratory and not beat-to-beat QRS variations. To the best of our knowledge, the present case is the first report of PVV on ECG in a patient with right-sided pneumothorax.

Second, the P-pulmonale and vertical P-wave axes were recognized in our case.

P-pulmonale, characterized by narrow and tall P-waves (≥ 2.5 mV in the inferior leads II, III, and aVF), is the most widely accepted diagnostic criteria for RA enlargement. P-pulmonale can be observed in many diseases associated with right-sided chambers’ strain; chronic lung disease [chronic obstructive pulmonary disease (COPD) and pulmonary emphysema], congenital heart disease (e.g., atrial septal defect and pulmonary stenosis), primary pulmonary hypertension, and right-sided heart failure associated with cor pulmonale. Thus, P-pulmonale has been considered a specific marker of right atrial overload (RAO) or wall thickness. However, a report on Swan-Ganz catheterization revealed no hemodynamic correlation with P-pulmonale in patients with chronic lung disease [14]. Another study using vectorcardiographic analysis demonstrated that patients with P-pulmonale were associated not only with RAO (49%) but also with left atrial overload (36%) [15]. Other studies also confirmed no correlation between P-pulmonale and RAO in patients with pressure and volume overload of the RA (only 8% in COPD, and 6% in atrial septal defect, respectively) [16, 17]. Besides RAO, the heart’s anatomical position with vertical orientation, left atrial overload, and electrolytic imbalance has been proposed as the causative factors to P-pulmonale [15, 18, 19]. Thus, P-pulmonale has a limited predictive value for RAO. We found no evidence of RA pressure burden in our case. Interestingly, the vertical P-wave axis, P-pulmonale, a straightened cardiac border in the RA, and the significantly depressed right diaphragm remained unchanged during respiration. All of them resolved immediately after releasing pneumothorax. The position and movement of the diaphragm can determine the position of the heart. The RA, which receives the openings of the superior and inferior venae cavae, is connected to the right diaphragm via the inferior vena cava and adjacent pericardium. During inspiration, the depressed diaphragm causes RA to stretch between the superior and inferior vena cavae, resulting in a vertical P-wave vector and the increased P-wave amplitude in the frontal plane. Therefore, the level of the diaphragm can be a critical determinant of P-wave axis/amplitude. Actually, the significant correlation between diaphragm levels and P-wave axis/amplitude was confirmed in patients with COPD, supporting this idea [20, 21]. Based on these findings, our case is highly likely to share the same pathophysiological mechanism as described above. Besides, positive intrathoracic pressure may be involved in the RA distortion because the RA is the most vulnerable structure to extracardiac pressure of all the cardiac chambers. Given that P-pulmonale and vertical P-wave axis have not been reported in patients with left-sided pneumothorax so far, these ECG findings can be considered specific to the right-sided pneumothorax.

Finally, these unique ECG findings may become an early indicator of a large/tension pneumothorax.

While pneumothorax can easily be susceptible to conversion to TPT with any eliciting events such as positive-pressure ventilation, percutaneous tracheostomy, or trauma, even PSP can be at risk of acquiring tension physiology [22, 23]. Moreover, suspicion or early recognition of subclinical TPT without any clinical evidence of tension physiology, such as hypoxemia, hypotension, and reflex tachycardia, remains extremely challenging.

Considering the vital signs at discharge, we deduce that our patient’s presentation with a large PSP (very close to a TPT) might have indicated a pre-shock state on admission. Therefore, the abnormal ECG findings recognized in our case can be considered to be specific to a large/tension pneumothorax rather than a simple small pneumothorax. From the viewpoint of the mechanism of ECG abnormalities, the P-pulmonale or vertical P-wave axis may be specific for a right-sided large PSP. Still, there remain concerns about a low diagnostic value for a secondary spontaneous pneumothorax with possibly similar findings due to existing lung disease. Besides, as shown in our case, PVV is highly likely to be a unique ECG sign of a large/tension pneumothorax, either left-or right-sided. The ECG manifestations may provide useful information to suspect a large pneumothorax or tension pneumothorax in emergent settings where ECGs are performed on patients with acute chest pain and dyspnea. However, the early and definitive diagnosis of pneumothorax requires a comprehensive approach that includes vital signs, physical examination, and multimodality imaging such as chest radiography and pleural ultrasonography. Further studies are needed to validate the usefulness of PVV on ECG as an early indicator of a large/tension pneumothorax.

Herein, we describe a case of a large right-sided PSP, presenting with characteristic findings on ECG. Although pneumothorax is a relatively common condition, the acquisition of tension physiology causes a potentially severe cardiopulmonary collapse, which is curable if treated timely with surgical intervention before hemodynamic deterioration. Therefore, emergency physicians and clinicians should be aware of the pneumothorax-associated ECG manifestations and bear in mind the suspicion of a large/tension pneumothorax in patients presenting with acute chest symptoms and a characteristic ECG finding of PVV.

## Supplementary Information

**Additional file 1.** (**a**: during inspiration, **b**: during expiration) The ECGs indicate increased heart rate, P-pulmonale (arrows), and vertical P-wave axis (arrowhead) irrespective of respiratory changes. Notably, all the R-wave voltages in the precordial leads (V4–6) are significantly augmented only during expiration (red arrows), fulfilling the Sokolow–Lyon ECG voltage criteria for left ventricular hypertrophy.

**Additional file 2.** (**a**: during inspiration, **b**: during expiration) Follow-up ECGs reveal the resolution of ECG abnormalities on admission. Notably, all the augmented R-wave voltages in the precordial leads (V4–6) recognized on initial ECG are not much induced even during expiration.

**Additional file 3.** (**a**: coronal view, **b**: axial view) Multiple bullae are recognized in both lung apexes (arrowheads). A large bulla with a maximum diameter of 46 × 49 mm is visible in the right lung apex (arrow).

**Additional file 4.** (**a**: during normal breathing; **b** during inspiration; **c** during expiration) Longer ECG recordings on admission during normal respiration reveal PVV of QRS complexes in V4–6. However, none of the longer ECG recordings during inspiration or expiration reveal similar finding of PVV.

## Data Availability

Data sharing is not applicable to this article as no datasets were generated or analyzed during the current study.

## Abbreviations

PSP: Primary spontaneous pneumothorax
TPT: Tension pneumothorax
CT: Computed tomography
ECG: Electrocardiogram
PVV: Phasic voltage variation
RA: Right atrium
COPD: Chronic obstructive pulmonary disease
RAO: Right atrial overload
