# Supplementary figures and images for: Electrocardiographic manifestations in a large right-sided pneumothorax
Source: BMC Pulm Med. 2021 Mar 23;21:101. doi: 10.1186/s12890-021-01470-1 (PMC7989373; doi:10.1186/s12890-021-01470-1)

## Slide 1
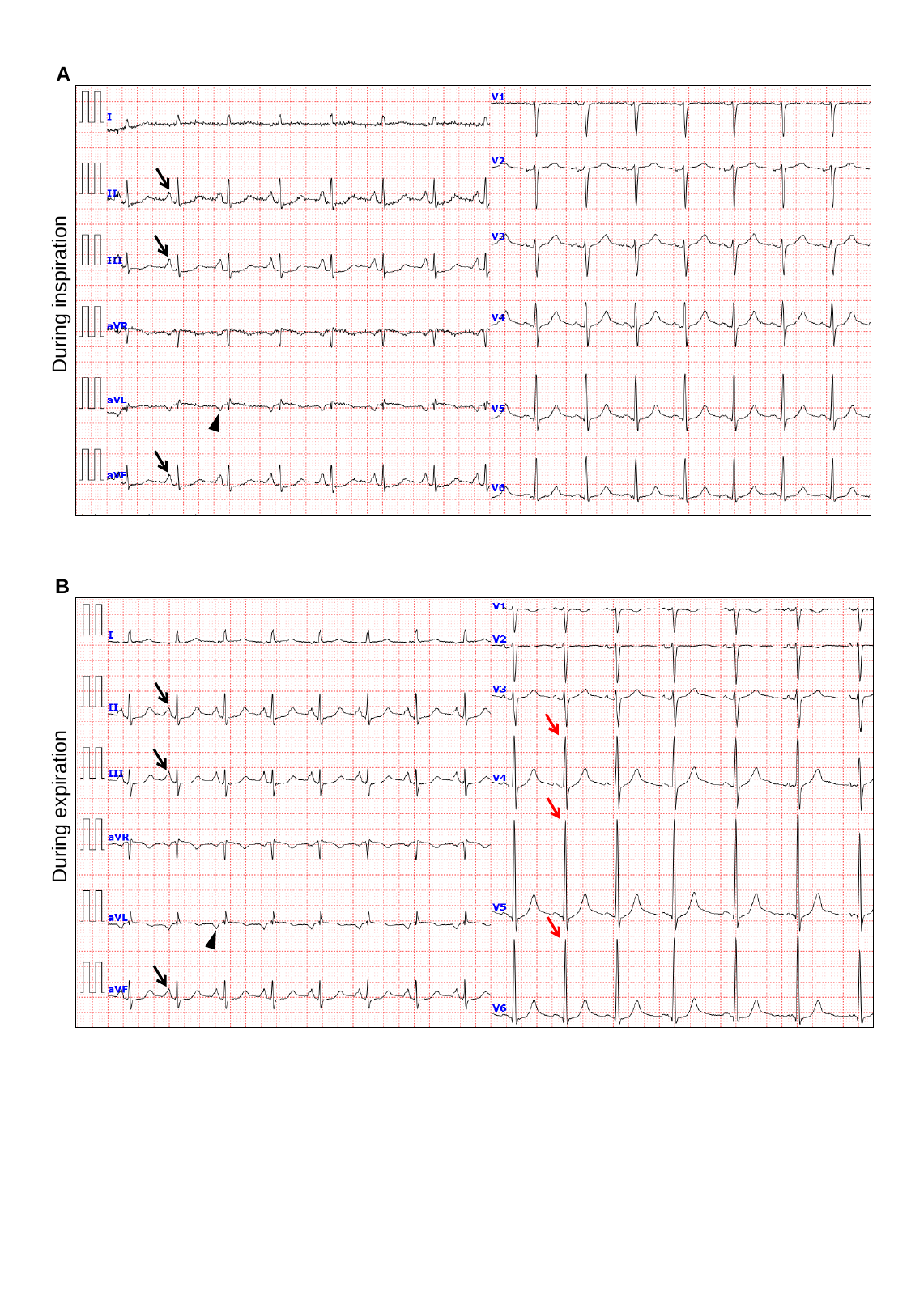

A
 During inspiration
B
 During expiration

Supplement: Supplementary file 1 — Additional file 1. (a: during inspiration, b: during expiration) The ECGs indicate increased heart rate, P-pulmonale (arrows), and vertical P-wave axis (arrowhead) irrespective of respiratory changes. Notably, all the R-wave voltages in the precordial leads (V4–6) are significantly augmented only during expiration (red arrows), fulfilling the Sokolow–Lyon ECG voltage criteria for left ventricular hypertrophy. [file 12890_2021_1470_MOESM1_ESM.pptx]

## Slide 1
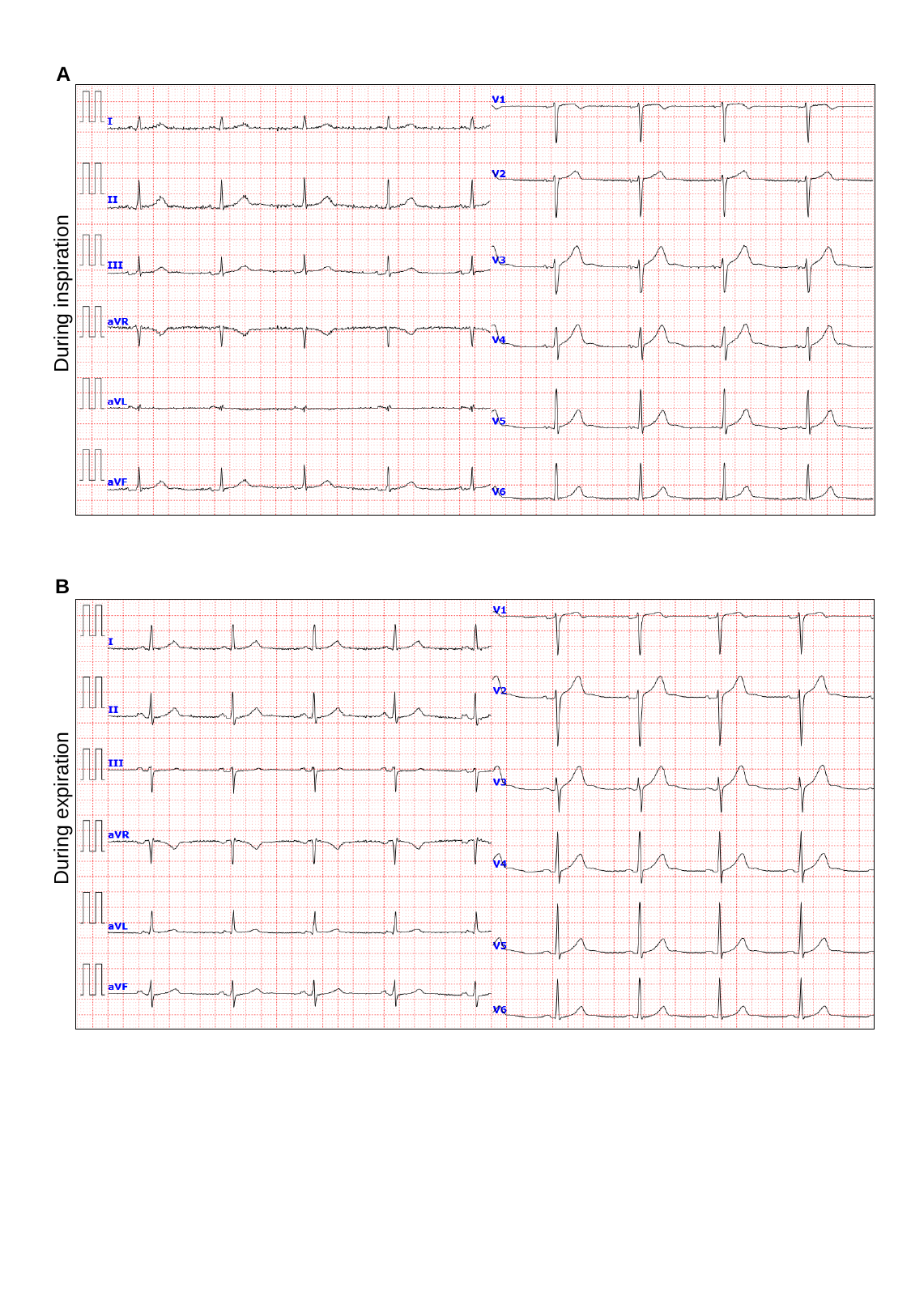

A
 During inspiration
B
 During expiration

Supplement: Supplementary file 2 — Additional file 2. (a: during inspiration, b: during expiration) Follow-up ECGs reveal the resolution of ECG abnormalities on admission. Notably, all the augmented R-wave voltages in the precordial leads (V4–6) recognized on initial ECG are not much induced even during expiration. [file 12890_2021_1470_MOESM2_ESM.pptx]

## Slide 1
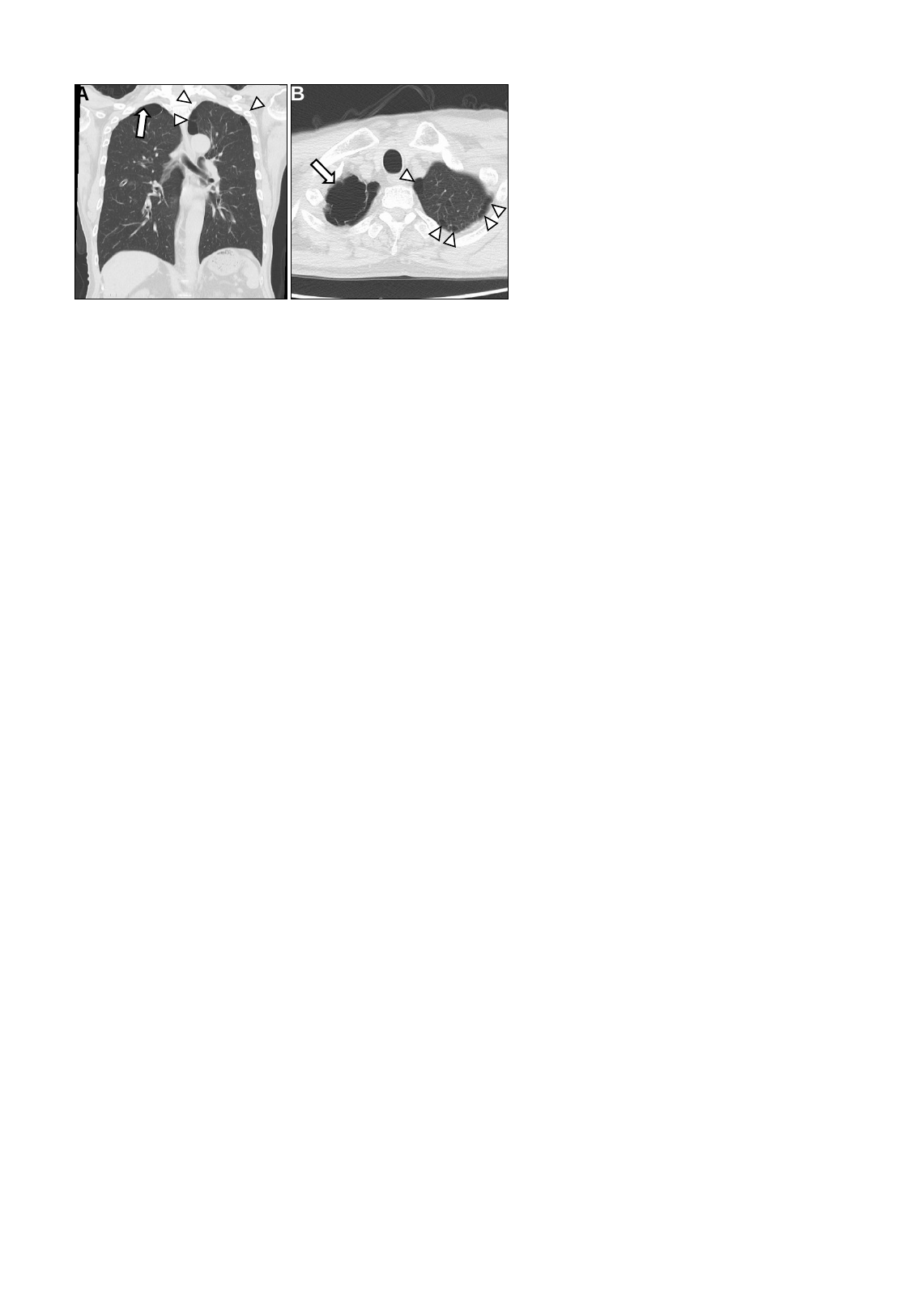

A
B

Supplement: Supplementary file 3 — Additional file 3. (a: coronal view, b: axial view) Multiple bullae are recognized in both lung apexes (arrowheads). A large bulla with a maximum diameter of 46 × 49 mm is visible in the right lung apex (arrow). [file 12890_2021_1470_MOESM3_ESM.pptx]

## Slide 1
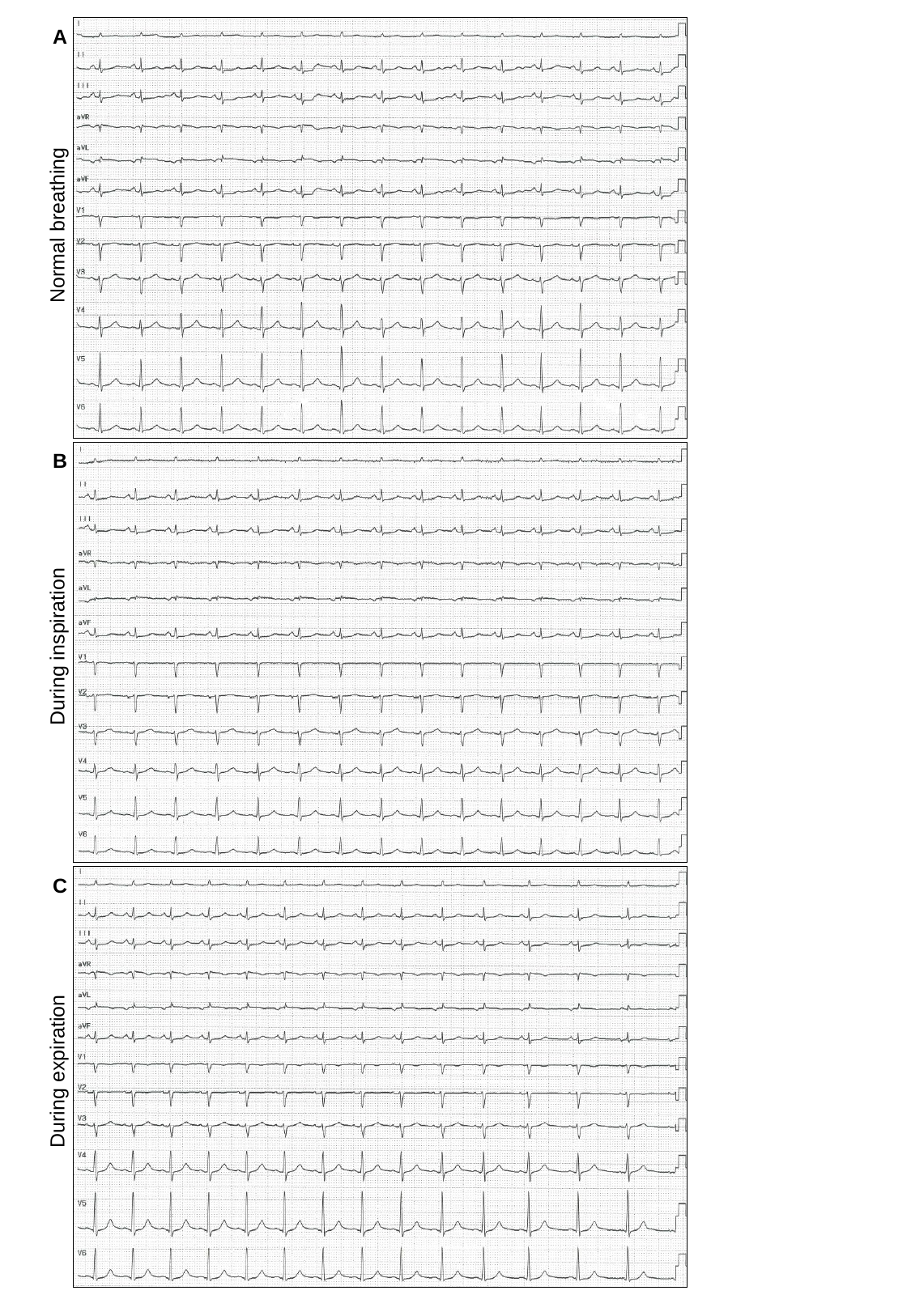

A
 Normal breathing
B
 During inspiration
C
 During expiration

Supplement: Supplementary file 4 — Additional file 4. (a: during normal breathing; b during inspiration; c during expiration) Longer ECG recordings on admission during normal respiration reveal PVV of QRS complexes in V4–6. However, none of the longer ECG recordings during inspiration or expiration reveal similar finding of PVV. [file 12890_2021_1470_MOESM4_ESM.pptx]
